# Non-Invasive Techniques Reveal Heifer Response to Fescue Endophyte Type in Grazing Studies

**DOI:** 10.3390/ani13142373

**Published:** 2023-07-21

**Authors:** Sanjok Poudel, John H. Fike, Lee Wright, Gabriel J. Pent

**Affiliations:** 1 School of Plant and Environmental Sciences, Virginia Tech, Blacksburg, VA 24061, USA; jfike@vt.edu; 2 Southwest Agriculture Research and Extension Center, Virginia Tech, Glade Spring, VA 24340, USA; lrite@vt.edu; 3 Shenandoah Valley Agriculture Research and Extension Center, Virginia Tech, Raphine, VA 24472, USA; gpent@vt.edu

**Keywords:** cortisol, fescue toxicosis, stress, vasoconstriction, thermography

## Abstract

**Simple Summary:**

This study compared the behavioral and physiological responses of heifers that grazed tall fescue infected with either a wild-type (WE) or a novel (non-toxic) endophyte (NE) using relatively non-invasive techniques such as hair cortisol, infrared cameras for extremities (ear, tail, and foot) temperatures, small loggers for intravaginal temperature, and remote imagery for monitoring animal behavior. Heifers on WE had cooler extremities temperatures and hotter intravaginal temperatures compared to those on NE. Hair cortisol levels were higher in heifers on WE compared to those on NE. Heifers on WE spent more time standing up and less time lying down during the daytime compared to those on NE. Overall, the findings indicate that replacing WE tall fescue with NE tall fescue can reduce heat load and corresponding stress in heifers, as indicated by changes in behavior, temperature, and cortisol levels. This study highlights the potential of non-invasive techniques such as thermographic imaging and hair cortisol analysis for assessing animal responses to stress in extensive grazing systems.

**Abstract:**

Cattle grazing tall fescue (*Schedonorus arundinaceous*) infected with wild-type endophytes (WE) leads to a syndrome commonly known as fescue toxicosis. Replacing WE tall fescue with a novel endophyte-infected (NE) tall fescue can mitigate this problem but adoption of this technology has been limited. This study measured and determined the physiological and behavioral responses of heifers that grazed either WE or NE tall fescue, utilizing relatively non-invasive techniques including hair cortisol, thermography (for extremity temperatures), small loggers for intravaginal temperature, and remote observation of in-field behavior. Heifers that grazed WE had greater (*p* < 0.0001) hair cortisol levels, lower extremity temperatures (*p* ≤ 0.0075), and 0.3–0.9 °C greater (*p* ≤ 0.02) intravaginal temperatures (particularly during the daytime) than heifers that grazed NE. From 1200 h–1700 h each day, heifers on WE pastures spent 1.5 more (*p* = 0.0003) hours standing up and 0.9 fewer (*p* = 0.0402) hours lying down than heifers on NE pastures. Differences (*p* = 0.0160) in ADG were small (0.1 kg d^−1^) and were only observed in the first year of these 8-week studies. However, even in the mild environment of the study site, grazing NE tall fescue provided clear welfare benefits as evidenced by heifer behavioral changes, temperature differentials, and hair cortisol levels. This study underscores the potential utility of non-invasive techniques, such as thermographic imaging and hair cortisol analysis, for evaluating animal responses to stress in extensive grazing systems.

## 1. Introduction

Tall fescue (*Schedonorus arundinaceous*) is the predominant cool-season perennial pasture forage found in the North–South transition zone of the USA, commonly known as the “fescue belt.” The plant harbors a wild-type endophytic fungus (WE; *Epichloe coenophiala*) that improves its persistence [1,2] but also produces a group of alkaloids that negatively impact grazing animals, causing fescue toxicosis [3,4]. The summer slump is a widespread condition associated with fescue toxicosis, which is observed during warm weather [5,6]. Common symptoms of summer slump include growth and retention of hair coats, heat stress, reduced intake and animal gain, and reproductive losses along with various other physiological and behavioral changes [7,8,9]. Heat stress due to fescue toxicosis is commonly observed during times of high ambient temperature (>32 °C) and relative humidity [10,11]. Scientists have discovered novel endophytes (NE) that produce few to no ergot alkaloids, which help maintain plant vigor and do not negatively impact animal performance [12]. Tall fescue infected with NE can reduce the effect of fescue toxicosis and improve livestock performance [8,9,13,14]. Although several studies have compared the growth and reproductive performance of cattle grazing WE and NE tall fescue [13,15], less work has been carried out to compare the behavioral and heat stress responses of cattle grazing these two pasture types. The lack of physiological and behavioral measures in grazing studies more likely reflects the challenge of gathering accurate data from animals in a grazing experiment. Hair cortisol, intravaginal temperature sensors, infrared thermography, and trail cameras are all relatively non-invasive methods that can provide accurate data on the temperature and stress response of grazing animals. However, these methods have received limited use as a means of assessing heat stress in animals, especially in quantifying the effects of toxic alkaloids in cattle grazing WE tall fescue. This study compared the behavioral and physiological responses of heifers grazing either NE or WE tall fescue using relatively non-invasive measurement techniques such as hair cortisol, thermography (for extremity temperatures), intravaginal temperatures, and remote observations of in-field behavior.

## 2. Materials and Methods

### 2.1. Study Site and Experimental Conditions

This study was conducted at Virginia Tech’s Southwest Virginia Agricultural Research and Extension Center (SWAREC) in Glade Spring, VA, USA, during the summers of 2020 (mid-July to early September) and 2021 (late June to late August). The SWAREC is located at 36°46′20.3″ N latitude and 81°48′25.8″ W longitude. The main soil types are Frederick silt loam and Timberville-Marbie complex with a slope ranging from 7 to 25 percent. The WE tall fescue pastures (cv ‘Kentucky-31’) used in this study were established about 25 years ago and have been grazed routinely by cattle and sheep. The NE tall fescue pastures (cv ‘Jesup MaxQ’) were established in 2007 and maintained with routine grazing. Three 0.6-ha pastures were used for each treatment. A soil test was carried out for each pasture prior to the study and was sent to a commercial soil lab for analysis. Based on a soil test recommendation, 70 kg ha^−1^ of nitrogen was applied in April of each year.

In 2020, twenty-four (24) fall-born Angus-cross heifers, 7 to 8 months old, from a university herd in Shenandoah Valley Agricultural Research and Extension Center, Raphine, VA, USA, were stratified based on body weight and hair coat color and randomly allocated to either of the two pasture treatments. In 2021, twenty-four (24) fall-born Angus or Angus-cross heifers, 7 to 8 months old, from a local producer’s herd were used following the same procedures. The mean initial body weight (BW) of heifers used for the study was 205 ± 3 kg in 2020 and 327 ± 5 kg in 2021. All heifers were weaned and raised on pastures consisting predominantly of ‘Kentucky-31’ tall fescue. For the study, both WE and NE fescue pastures were stocked with heifers for an 8-week grazing period each year. Heifers within each treatment were rotationally stocked among three pastures based on forage availability determined by visual observation of stand height. Animals were provided with supplemental minerals (Purina^®^ Wind and Rain^®^ All Season, Purina Animal Nutrition LLC., Arden Hills, MN, USA) and clean drinking water ad libitum.

### 2.2. Weather Data

Ambient temperature (AT), maximum and minimum temperatures, relative humidity (RH), and rainfall data for the research site were obtained from Virginia Tech WeatherSTEM Data Mining Tool (http://vt-arec.weatherstem.com, accessed on 16 January 2022) for the entire study period in both years. Additionally, the daily average temperature humidity index (THI) was calculated using the AT and RH data for the entire study period. Hourly AT and RH data were downloaded for the specific dates when intravaginal temperature and animal behavior data were recorded, and average THI was calculated by the hour using the equation developed by Mader et al. [16]:

$$\mathrm{THI}=\left(0.8\times \mathrm{AT}\right)+\left(\frac{\mathrm{RH}}{100}\right)\times \left(\mathrm{AT}-14.4\right)]+46.4.$$

### 2.3. Forage Analysis

The total ergot alkaloid (TEA) concentrations in fescues from the experimental pastures were assessed in 2019 and 2020 by collecting grab samples of tall fescue plants from 50 random locations within each pasture. These samples were combined to form a composite sample that accurately represents the entire field. Samples were then placed in plastic bags on ice in the field and then stored at −70 °C until freeze-drying. After freeze drying, samples were initially ground using a Wiley mill (Thomas Scientific, Swedesboro, NJ, USA) with a 2-mm screen, followed by further grinding using a cyclone mill (UDY Corporation, Fort Collins, CO, USA) with a 1-mm screen to achieve the desired particle size. Samples were analyzed for TEA concentrations using a commercial ELISA test kit (Catalog no. ENDO899-96p; Agrinostics Ltd., Watkinsville, GA, USA).

The botanical composition of each pasture was determined by visual estimation before introducing the heifers into the pasture. The composition was calculated as the proportion of each observed species within 0.25-m^2^ quadrats sampled at 10 random locations within each treatment pasture. Available forage biomass within each pasture was estimated by clipping eight quadrats (0.25 m^2^) to a 5 cm height before introducing the heifers into the pasture. Clipped samples were dried at 60 °C for 72 h and dry weights were used to calculate estimates of available forage biomass. These dried samples were also used for nutritive value estimation. The samples underwent grinding in a Wiley mill (Thomas Scientific, Swedesboro, NJ, USA) to pass through a 2-mm screen, followed by further grinding in a cyclone mill (UDY Corporation, Fort Collins, CO, USA) to pass through a 1-mm screen. Ground samples were scanned with a near-infrared spectrophotometer (DS2500F using ISIScan Nova v. 8.0.6.2, Foss North America, Eden Prairie, MN, USA). Estimates of crude protein (CP), acid detergent fiber (ADF), and neutral detergent fiber (NDF) were made using the 2022 Grass Hay calibration provided and licensed by the NIRS Forage and Feed Consortium [17].

### 2.4. Animal Gain and Hair Retention Score

Heifers’ BW was measured at the beginning (day 1), mid- (day 28), and end (day 56) of the study in the morning between 0900–1100 h without an overnight fast. Heifer average daily gain (ADG) was calculated by dividing the change in body weight (BW) by the number of days the animal grazed on the pasture. Hair retention scores were determined using a 5-point scale [18]. A score of 5 indicates that an animal has retained its complete winter coat and exhibits no evidence of shedding while a score of 1 indicates that the animal has completely shed its winter coat and exhibits a slick summer hair coat. Data for hair retention scores were recorded at the beginning (day 1), mid- (day 28), and end (day 56) of the study for both years. A single evaluator assigned hair retention scores for individual heifers for each data collection date throughout the study.

### 2.5. Extremity and Intravaginal Temperatures

Thermal images of the body extremities (i.e., ear, front left foot, and tail) were taken at the middle (day 28) and end (day 56) of the study using an FLIR T630SC thermal camera (Teledyne FLIR LLC., Santa Barbara, CA, USA) in the morning between 0900h and 1100 h. Animals were placed in a holding area and passed one by one into a covered and shaded handling chute where thermal images were captured. A distance of 1 m from the extremity was maintained while capturing images. Captured images were processed with FLIR Research IR Max software (Version 4.40.9.30) to determine the average temperature of the area of interest within the image. For foot images, the pastern, coronary band, perioplic band, heel, wall, and toes were selected within the image as an area of interest to determine the average temperature.

Intravaginal temperatures of heifers were collected with small temperature loggers (Star Oddi Data Storage Tag (DST) micro-T temperature logger, Star Oddi, Garðabær, Iceland) secured inside blank controlled internal drug release (CIDR) devices (Eazi-Breed, Zoetis, Parsipanny, NJ, USA). These temperature loggers were placed into the vaginas of heifers twice for two consecutive days at 4-week intervals and collected temperature data every 10 min. Data were collected twice in the middle (days 26 and 27) and end (days 54 and 55) of the study. Data were later retrieved using a communication box attached to a computer using Mercury software (Star Oddi, Garðabær, Iceland).

### 2.6. Hair and Blood Sample Collection and Cortisol Analysis

Hair and blood samples from each heifer were collected at the beginning of the experiment (day 0) and were used as baseline measures of cortisol. A 15 cm × 15 cm area on the rump region of the heifers was clipped as closely to the skin as possible using an electric clipper (900cl Cordless Clipper with Eagle 30 Small Clipper Blade Set, Premier 1 Supplies, Washington, IA, USA). The same site was sampled again in the middle (day 28) and at the end (day 56) of the trial. Hair samples were wrapped in aluminum foil and stored at room temperature until analysis. Blood samples were collected from the coccygeal vein using an 18G x 1” needle (Greiner Bio-One North America Inc., Monroe, NC, USA) and a vacutainer tube containing potassium-EDTA as an anticoagulant (Becton, Dickinson and Company, Franklin Lakes, NJ, USA). After collection, the samples were placed on ice and later separated into plasma through centrifugation at 3400× *g* for 15 min at room temperature. The resulting plasma samples were then stored at −70 °C until further analysis.

Hair and plasma cortisol extraction was performed according to the method described by Poudel et al. [19]. Cortisol concentration from hair and blood sample extractions was quantified with a commercial salivary cortisol ELISA kit (Cortisol ELISA Kit Item No. 500360, Cayman Chemical, MI, USA) according to the manufacturer’s instructions. The inter-assay CV and intra-assay CV for the ELISA test kit were 16.4% and 9.9%, respectively.

### 2.7. Animal Behavior Data

For behavioral data, time-lapse images were collected using Moultrie D-500 trail cameras (EBSCO Industries, Inc., Birmingham, AL, USA). Within each treatment pasture, two cameras were set up to visually encompass the entire pasture at the middle (days 26 and 27) and end (days 54 and 55) of the study. Cameras were set to capture images at 1-min intervals from 0800 h to 2100 h. The photos were subsequently processed manually. For every image, the number of animals engaged in a given behavior (grazing, standing up, lying, drinking water, eating minerals) was recorded manually by reviewing each image. The minutes spent engaged in each behavior were summed and used to calculate the percent of time spent performing each activity. Each day was further divided into morning (0800–1200 h), afternoon (1200–1700 h), and evening (1700–2100 h) periods to determine how pasture type affected animal behavior at different times of the day. Behavioral data were only collected in year one since cameras installed during year two stopped working due to unforeseen technical issues and images were not retrievable.

### 2.8. Statistical Analysis

Statistical analyses were conducted using SAS Studio, v9.4 (SAS Inst., Cary, NC, USA). The study was analyzed as a completely randomized design with a heifer treated as an experimental unit for all animal response variables. A mixed model ANOVA test was conducted using PROC MIXED to evaluate the effects of treatment. Year was included in the model as a random effect. The baseline measures of plasma and hair cortisol levels were also subjected to statistical analysis to ascertain potential differences between the treatments. A repeated-measures analysis by period was utilized to examine ADG, hair retention score, cortisol measures, extremity temperature, and intravaginal temperature data with a standard variance-covariance structure. LS-means and Tukey’s adjusted differences were calculated, and significance was defined as *p* < 0.05, with trends considered at *p* < 0.10.

## 3. Results

### 3.1. Weather Data and Forage Measures

**Figure 1:**
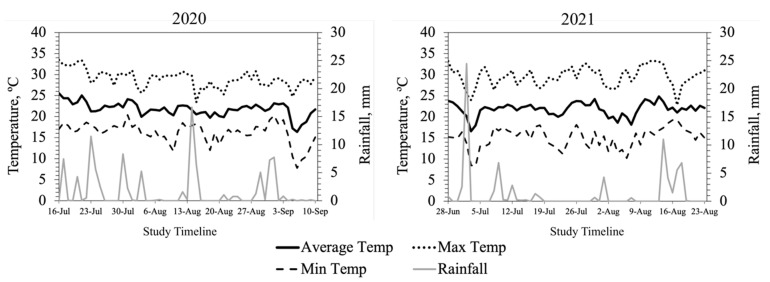
Daily average, minimum, and maximum temperatures (°C) and precipitation (mm) throughout the study period at Southwest Agriculture Research and Extension Center, Glade Spring, VA, USA.

**Figure 2:**
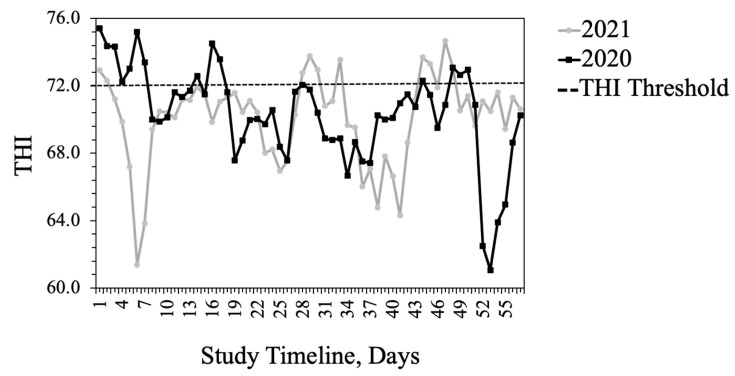
Daily average temperature-humidity index (THI) throughout the study period at Southwest Agriculture Research and Extension Center, Glade Spring, VA, USA.

**Figure 3:**
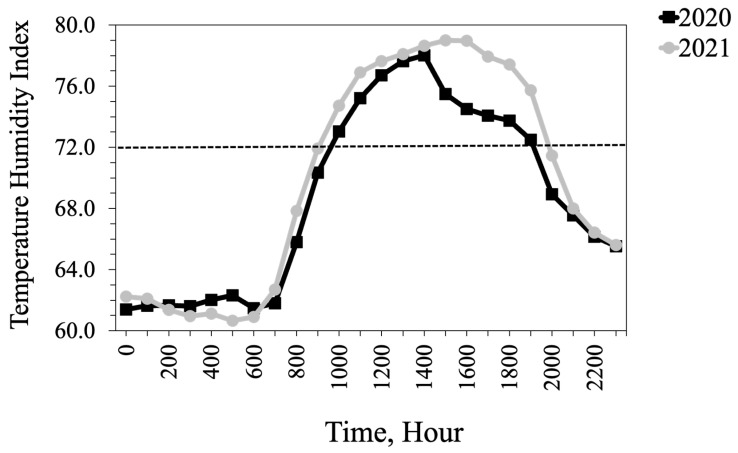
Average temperature-humidity index (THI) by hours during the intravaginal temperature and heifers’ behavior data collection dates for 2020 and 2021 at Southwest Agriculture Research and Extension Center, Glade Spring, VA, USA.

The average daily AT was similar across both summers at the study site (Figure 1). More precipitation fell throughout 2020 than in 2021 (9.9 cm vs. 7.7 cm). The mean THI was 70.4 in 2020 and 70.0 in 2021. In 2020, the maximum THI was 75.4 and the average daily THI exceeded the thermoneutral zone for cattle (THI = 72) for 15 days. In 2021, the maximum THI was 75.7 but the average THI exceeded the thermoneutral zone for 10 days (Figure 2). During the time of intravaginal temperature and animal behavior data collection, THI was above the thermoneutral zone of cattle (72) during the late morning and afternoon hours (1000 h to 1900 h; Figure 3). The average TEA concentration of WE tall fescue was 1554 ppb while the average TEA concentration of NE tall fescue was 398 ppb.

The dominant vegetation in both the WE and NE pastures was tall fescue, with no significant difference (*p* = 0.4404) observed in tall fescue composition between the two treatment pastures (Table 1). Other common species observed in both pastures included orchardgrass (*Dactylis glomerata*), quackgrass (*Elytrigia repens*), smooth bromegrass (*Bromus inermis*), and several leguminous species such as white clover (*Trifolium repens*), red clover (*T. pratense*), and hairy vetch (*Vicia villosa*). Overall forage biomass did not differ (*p* = 0.8041; Table 2) between treatments. For CP and ADF, there were year × treatment interactions (*p* ≤ 0.0352). In 2020, NE tall fescue pastures had greater CP (*p* = 0.0117) and lower ADF (*p* = 0.0013) concentrations than WE tall fescue pastures. Across both years, NE tall fescue pastures had lower NDF (*p* = 0.0266) concentrations than WE tall fescue pastures. Forage CP content was greater (*p* = 0.0003) during the first year compared to the second year of the study.

### 3.2. Animal Gain and Hair Retention Score

Heifers had greater (*p* < 0.0001) ADG in 2021 than in 2020. In 2020, heifers that grazed NE tall fescue had greater (*p* = 0.0160) ADG than those on the WE treatment (0.22 vs. 0.12 kg d^−1^; Table 3). However, in 2021, heifer ADG did not differ (*p* = 0.9623) by pasture treatment. Despite the year-to-year variation, no year × treatment interaction was observed (*p* = 0.2756). Heifers that grazed NE fescue had lower (*p* = 0.0029) hair retention scores compared to heifers that grazed WE tall fescue (Table 3).

### 3.3. Extremity and Intravaginal Temperature

**Figure 4:**
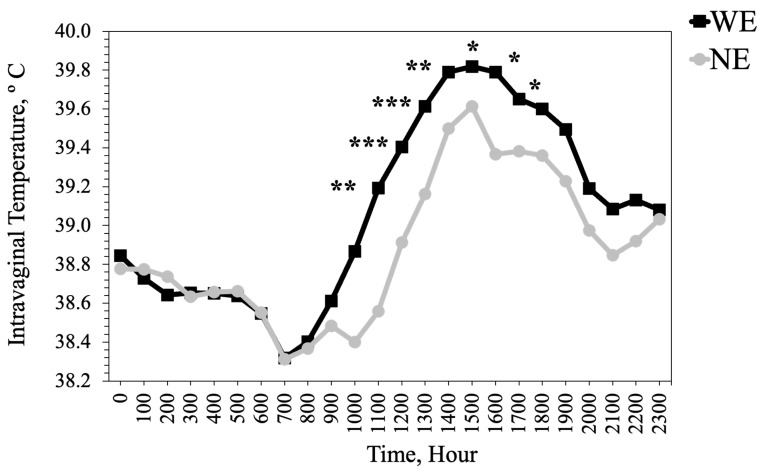
Comparison of mean vaginal temperatures (SE- 0.10) of heifers that grazed either toxic (WE) or novel endophyte (NE) infected tall fescue by the hour of the day. Level of significance: * *p* < 0.05, ** *p* < 0.01, *** *p* < 0.001.

Ear, tail, and hoof temperatures were lower (*p* ≤ 0.0075) for heifers that grazed WE tall fescue than heifers that grazed NE tall fescue (Table 4). Over the two summers, heifers that grazed WE tall fescue had 0.2–0.5 °C hotter (*p* ≤ 0.02) intravaginal temperatures between 1100 h and 1700 h than heifers that grazed NE tall fescue (Figure 4).

### 3.4. Plasma and Hair Cortisol Levels

There was no difference in baseline measures of plasma and hair cortisol levels between treatments (*p* > 0.7389). Plasma cortisol levels did not differ (*p* = 0.6579) between treatments. In contrast, hair cortisol levels were greater (*p* < 0.0001) for heifers that grazed WE pastures compared to heifers that grazed NE pastures across both summers (Table 5).

### 3.5. Animal Behavior

Heifers that grazed WE and NE fescue had distinctly different behavioral patterns, especially during the daytime. From 1200 h to 1700 h each day, heifers on WE pasture spent 1.5 more (*p* = 0.0003) hours standing up and 0.9 fewer (*p* = 0.0402) hours lying down compared to heifers that grazed NE pastures (Table 6). Overall, heifers that grazed WE fescue spent 36.7% of observation time grazing, 40.9% of observation time standing up, and 22.1% of observation time lying. Heifers that grazed NE fescue spent similar (37.3%) time grazing, less time standing up (33.7%), and more time lying down (28.8% of observation time).

## 4. Discussion

The concentration of TEA in WE tall fescue ranged from 1234 to 1565 ppb; pastures were considered to be toxic enough to induce symptoms of fescue toxicosis [20]. Animals grazing WE tall fescue may display visible signs of fescue toxicosis when TEA concentrations of a pasture are greater than 400 ppb [21,22]. As the novel endophytes within NE tall fescue produce minimal or no toxic alkaloids, animals grazing these varieties display no signs of fescue toxicosis [12]. Although the NE pastures had alkaloid levels at the threshold for inducing symptoms (perhaps due to WE tall fescue incursion), clear differences in physiological and behavioral measures as a function of toxin consumption were observed in this study. Forage productivity was comparable among WE and NE tall fescue across both years. Past studies have shown that NE and WE tall fescue respond similarly in a given environment [23,24,25] and persist under similar grazing conditions [26]. Novel endophyte within NE tall fescue supports plant persistence and vigor similar to that observed for WE tall fescue plants but without any deleterious effects on livestock [26].

Many studies indicate that livestock grazing tall fescue with WE have substantial production losses compared to non-toxic tall fescue [8,27,28]. Such differences were less pronounced for the heifers grazing WE fescue in this study. Heifers on WE tall fescue had lower ADG than heifers grazing NE tall fescue, but only in 2020. In 2020, WE tall fescue had lower forage quality characteristics compared to NE tall fescue and this may partially explain the year-to-year differences in heifer performance. Also, differences in weather patterns between the two years of the study and the severity of fescue toxicosis are highly dependent on environmental conditions [10]. In the cooler climate of Illinois, pregnant cows did not differ in gain on WE and NE fescue; however, physiological responses (respiration rate, hair coat, and prolactin levels) differed and were benefitted by grazing NE [15]. The average THI exceeded the thermoneutral zone for cattle (72) for 15 days in 2020, largely at the beginning of the study, when heifers had likely experienced the added stress of shipping some distance. In 2021, heifers were provided by a local producer and THI exceeded thermoneutral for 10 days primarily toward the end of the study.

Differences in the origin of the heifers likely contributed to differential animal gain between years. Heifers in 2020 were transported from a university herd located in the Shenandoah Valley, located several hours to the north of the study site. Shipping causes stress [29] that can be reflected in transient elevations of plasma cortisol [30]. Moreover, young cattle do not tolerate stress as well [31] and the heifers used for the study in 2021 were older and heavier than those used in 2020 (327 kg compared to 205 kg, respectively)

Decreased weight gain in heifers grazing WE tall fescue under increased environmental temperature may be a result of reduced DMI in stressed animals [32] as has been observed in past studies comparing animal gains on NE and WE tall fescue [8,33]. Lowering DMI is a typical strategy for maintaining homeostasis when animals experience heat stress [34,35]. This reduces metabolic heat from fermentation and digestion, which would further exacerbate the elevated core temperatures associated with vasoconstriction. The short duration of this study (8 weeks) likely further limited our ability to see a substantial treatment effect for this parameter. However, whether or not the environment was sufficiently stressful or the study was long enough to drive larger differences in gain, there were clear physiological and behavioral differences in heifers between treatments. The ADG was well below target gains for this class of heifers in both years of the study and this may be attributed to various factors, such as post-weaning stress and other unaccounted-for variables. The body weights were taken without an overnight fasting and differences in feed or water intake could also explain the unexpected low BW gains in such a short-term study.

Heifers on WE tall fescue shed less of their winter hair coats compared to heifers on NE, and this is associated with low prolactin levels in cattle exhibiting symptoms of fescue toxicosis [6,36]. The follicular cycle in cattle is regulated by the prolactin hormone secreted by the pituitary gland [37], and the prolactin level increases as a function of increased day length [38]. However, ergovaline acts as a dopamine agonist that mimics the binding of dopamine and blocks dopamine D2 receptors, thus reducing the synthesis and secretion of prolactin from the pituitary gland [39]. As serum prolactin levels remain low, this both inhibits shedding and supports the uncontrolled growth of new hair during the summer [8,37,38], resulting in a greater hair retention score of cattle on WE tall fescue compared to non-toxic tall fescue.

Heifers that grazed WE tall fescue had cooler extremities temperatures and hotter intravaginal temperatures, especially during periods of the day when THI was above the thermoneutral zone of cattle. Higher intravaginal and lower extremity temperatures in heifers that grazed WE tall fescue compared to heifers that grazed NE tall fescue reflect the vasoconstrictive effects of toxic alkaloids within the WE tall fescue. Smaller vessel diameter restricts blood flow to extremities and heat dissipation from the animal body [39,40,41], resulting in cooler ear, tail, and hoof temperatures in animals experiencing fescue toxicosis. Various studies have reported decreased extremity temperature in animals in response to toxic ergot alkaloids consumption [42,43] and thermography has been used to study testicular temperatures for bulls on fescue [44]. However, thermography has been very limitedly used in studies to measure extremity temperature to quantify heat stress in response to consuming WE tall fescue [45] and this could be a useful tool for rapid, non-invasive screening. Hotter extremity temperature in heifers on NE tall fescue reflects improved blood flow, which would allow for improved heat dissipation from the body extremities. The restricted dissipation of body heat results in increased internal body temperatures, thereby causing heat stress in animals consuming WE tall fescue [32,40,46].

Cortisol is a stress hormone released by the adrenal cortex in response to a stressor such as heat stress. Animals exposed to fescue alkaloids can exhibit increased circulating cortisol, although this response has been variable. Cattle infused with ergotamine have increased cortisol levels [47,48] but no cortisol response to alkaloids was observed by Aldrich et al. [39] or Looper et al. [49].

Blood is commonly used as the tissue of choice for measuring cortisol levels but this is problematic, as animals very quickly elevate plasma cortisol in response to acute stress [50]. Typical blood sampling procedures require capturing and restraining animals that can induce acute stress, leading to rapid increases in blood cortisol levels [51]. This stress-induced response may introduce variability in the data and could explain the absence of observed differences in plasma cortisol levels among treatments in our study.

Because of these challenges, hair cortisol may be a better measure with which to understand the level of stress experienced by animals in grazing situations [52,53,54]. Hair may be a good material to assay because cortisol from blood is diffused into the hair at the follicle and accumulates over time. Hair cortisol is a reliable indicator of long-term chronic stress levels in animals over extended periods [55], unaffected by activities like handling or restraining [52,56]. Previous studies have demonstrated a positive association between hair cortisol levels and stressors such as heat stress, handling, and transport [53,57]. Therefore, hair cortisol holds promise as a reliable method to assess long-term chronic heat stress, exposure to wild-type tall fescue, or both. For this study, measuring cortisol in hair provided a novel approach to assessing stress levels of heifers grazing different fescue types. The method was relatively less invasive and not sensitive to short-term animal disturbance—important in this case as animals were moved some distance from the field to handling facilities. Moreover, the sampling of hair is simple, and the samples can be stored at room temperature for a long time [55]. The lower cortisol levels in the hair of animals that grazed NE tall fescue provide a physiological marker of lower stress in these heifers compared to heifers grazing WE. These animals also exhibited lower body and greater extremity temperatures and altered time budgets compared to heifers that grazed WE fescue. Parsing the direct effects of TEA consumption and heat stress on hair cortisol was not possible in this study, as the heifers that consumed greater levels of alkaloids also had greater heat stress levels due to increased vasoconstriction.

Heat stress due to fescue toxicosis can severely impact the behavior of grazing animals, and these alterations in animal behavior severely impact their overall productivity [32,58]. Various behavioral changes, such as seeking shade, forming wallows around water troughs and in shaded areas, and spending less time grazing, are exhibited by animals experiencing fescue toxicosis [6,36]. We acknowledge that there was a loss of trail camera data for year two due to unforeseen technical issues, which limited our ability to obtain behavioral observations consistently across both years. However, it is important to note that timelapse trail cameras have been utilized as a reliable method with which to monitor animal behavior, including those examining grazing patterns, social interactions, and activity levels [19,59]. Their findings demonstrated the applicability and effectiveness of this technique for assessing behavioral responses.

Observed changes in heifer standing up and lying times likely reflect the diminished thermoregulatory ability of the heifers on WE in response to ambient temperatures [39]. Heifers grazing on WE tall fescue may have adopted a standing posture as a strategy to enhance airflow and improve convective heat loss from their bodies, potentially reducing the heat load they experienced [60]. Higher standing time is an adaptive mechanism of animals to limit heat gain from the ground surface and enhance heat loss through improved airflow to the body surface [61]. The THI during the afternoon hours, when behavior data were recorded, was above the thermoneutral zone of cattle, thus exacerbating the effects of fescue toxicosis on these animals.

## 5. Conclusions

Tall fescue is the predominant pasture forage in the southeastern U.S. The wild-type endophyte within the tall fescue plant produces toxic alkaloids that act as a vasoconstrictor, thus increasing heat stress and resulting in significant production and reproductive losses in the beef industry. The use of NE tall fescue may be a viable option for reducing the effects of fescue toxicosis, which could ultimately result in improved profitability for producers. The findings revealed that heifers grazing on NE tall fescue exhibited reduced heat stress compared to those grazing on WE tall fescue. This was evidenced by lower extremity temperatures, slightly higher intravaginal temperatures, and lower hair cortisol levels observed in heifers on NE pastures. Moreover, heifers on NE grass demonstrated more favorable behavioral patterns, with increased time spent lying down during the daytime. The non-invasive techniques used in this study for measuring stress, such as thermographic imaging and hair cortisol analysis, were particularly effective for use with free-ranging cattle and may have applications in other livestock management contexts, such as identifying animals that are experiencing heat stress or other forms of chronic stress.

Overall, this research provides valuable evidence supporting the adoption of NE tall fescue as an effective strategy to reduce the effects of fescue toxicosis in livestock. The findings have implications for livestock management practices, particularly in fescue-based systems, and shed light on the broader potential of non-invasive techniques in evaluating animal responses to stressors. Further research and long-term studies are warranted to validate these findings and explore additional benefits of NE tall fescue in various livestock management contexts.

## Figures and Tables

**Table 1:** Botanical composition of toxic (WE) and novel (NE) endophyte-infected tall fescue pastures during the summers of 2020 and 2021 at Southwest Agriculture Research and Extension Center, Glade Spring, VA, USA.

Year	Treatments ^1^			
	WE	NE	SE	*p*-Value
Tall Fescue (*Schedonorus phoenix*), %				
2020	73.4	77.5	3.38	0.3979
2021	75.6	76.1	1.97	0.8420
Average	74.5	76.8	1.83	0.4404
Orchardgrass (*Dactylis glomerata*), %				
2020	9.6	6.0	2.04	0.2121
2021	8.1	7.2	1.18	0.5965
Average	8.9	6.6	1.10	0.1951
Quack Grass (*Elymus repens*), %				
2020	6.2	6.6	2.52	0.8964
2021	2.6	3.8	1.28	0.5013
Average	4.4	5.2	1.29	0.6083
Kentucky Bluegrass (*Poa pratensis*), %				
2020	2.9	5.5	1.36	0.1877
2021	2.6	1.7	0.87	0.4707
Average	2.7	3.6	0.77	0.6438
Legumes, %				
2020	0.3	0.7	0.36	0.5223
2021	3.6	3.6	1.42	1.0000
Average	1.9	2.1	0.88	0.9133
Other Broadleaf, %				
2020	5.5	3.7	1.42	0.3759
2021	7.7	7.7	1.07	1.0000
Average	6.6	5.7	0.88	0.5536

^1^ Treatments: WE—Toxic endophyte-Infected tall fescue; NE—Novel endophyte-infected tall fescue.

**Table 2:** Available forage biomass and quality characteristics of toxic (WE) and novel (NE) endophyte-infected tall fescue pastures during the summers of 2020 and 2021 at Southwest Agriculture Research and Extension Center, Glade Spring, VA, USA.

Year	Treatments ^1^			
	WE	NE	SE	*p*-Value
Forage Biomass, kg ha^−1^				
2020	3010	3170	2350	0.6432
2021	2650	2320	1410	0.1072
Average	2830	2740	2539	0.8041
Crude Protein ^2^, %				
2020	13.8	15.8	0.54	0.0117
2021	12.8	12.1	0.41	0.2532
Acid Detergent Fiber ^2^, %				
2020	35.5	32.8	0.56	0.0013
2021	34.2	33.5	0.53	0.3726
Neutral Detergent Fiber, %				
2020	64.5	60.3	0.89	0.0016
2021	63.4	62.2	1.17	0.4559
Average	63.9	61.2	0.85	0.0266

^1^ Treatments: WE—Toxic endophyte-Infected tall fescue; NE—Novel endophyte-infected tall fescue. ^2^ Significant year × treatment interaction (*p*-value: Crude Protein = 0.0352; Acid Detergent fiber = 0.0155).

**Table 3:** Average daily gain (ADG; kg d^−1^) and hair retention score of heifers that grazed either toxic (WE) or novel (NE) endophyte-infected tall fescue during the summers of 2020 and 2021.

Year	Treatments ^1^			
	WE	NE	SE	*p*-Value
ADG ^2^, kg d^−1^				
2020	0.12	0.22	0.028	0.0160
2021	0.49	0.49	0.050	0.9623
Average	0.31	0.36	0.031	0.2377
Hair Retention Score ^3^				
2020	3.3	2.8	0.15	0.0158
2021	3.0	2.5	0.13	0.0283
Average	3.1	2.7	0.11	0.0029

^1^ Treatments: WE toxic endophyte-infected tall fescue; NE novel endophyte-infected tall fescue; Twelve fall-born Angus or Angus cross heifers were assigned to each pasture treatment. ^2^ ADG—Average daily gain. ^3^ Hair retention score based on a 5-point scale. Scoring for the 5-point scale: 5 = complete retention of winter coat; 4 = approximately 75% retention of winter coat; 3 = approximately 50% retention of winter coat; 2 = approximately 25% retention of winter coat; 1 = no retention of winter coat.

**Table 4:** Extremity temperatures (°C) of heifers that grazed either toxic (WE) or novel (NE) endophyte-infected tall fescue during the summers of 2020 and 2021.

Year	Treatments ^1^			
	WE	NE	SE	*p*-Value
Ear Skin Temperature ^2^, °C				
2020	29.5	30.6	0.45	0.0770
2021	29.6	31.8	0.25	<0.001
Average	29.5	30.2	0.30	0.0001
Hoof Surface Temperature ^2^, °C				
2020	24.9	26.3	0.66	0.1447
2021	27.8	29.6	0.21	<0.001
Average	26.4	28.0	0.41	0.0075
Tail Skin Temperature ^2^, °C				
2020	26.0	25.5	0.35	0.3226
2021	27.2	28.7	0.25	0.0001
Average	26.4	27.4	0.25	0.0058

^1^ Treatments: WE toxic endophyte-infected tall fescue; NE novel endophyte-infected tall fescue; Twelve fall-born Angus or Angus cross heifers assigned to each pasture treatment. ^2^ Extremity temperatures were determined using a FLIR T630SC thermal camera (Teledyne FLIR LLC, Santa Barbara, CA, USA).

**Table 5:** Plasma cortisol (ng ml^−1^) and hair cortisol (pg mg^−1^) concentration of heifers that grazed either toxic (WE) or novel (NE) endophyte-infected tall fescue during the summers of 2020 and 2021.

Year	Treatments ^1^			
	WE	NE	SE	*p*-Value
Plasma Cortisol, ng mL^−1^				
2020	4.6	4.0	0.19	0.0356
2021	10.3	9.2	1.31	0.5699
Average	7.5	6.6	1.32	0.6579
Hair Cortisol, pg mg^−1^				
2020	8.1	6.3	0.40	0.0009
2021	5.0	3.2	0.42	0.0033
Average	6.6	4.7	0.29	<0.0001

^1^ Treatments: WE toxic endophyte-infected tall fescue; NE novel endophyte-infected tall fescue; Twelve fall-born Angus or Angus cross heifers were assigned to each pasture treatment.

**Table 6:** Percent of time spent by heifers under different behavior categories on either toxic (WE) or novel (NE) endophyte-infected tall fescue pastures during the summer of 2020.

Behavior Category ^2^	Period	Treatments ^1^			
		WE	NE	SE	*p*-Value
Grazing (%)	Morning (700–1200)	30.0	22.0	2.98	0.1281
	Midday (1200–1700)	19.3	31.4	4.80	0.1496
	Evening (1700–2100)	60.9	58.5	6.76	0.8105
	Overall	36.7	37.3	1.83	0.8480
Standing Up (%)	Morning (700–1200)	44.2	42.0	9.79	0.8836
	Midday (1200–1700)	63.4	33.5	1.77	0.0003
	Evening (1700–2100)	15.0	25.4	7.89	0.4027
	Overall	40.9	33.7	4.78	0.3462
Lying (%)	Morning (700–1200)	25.1	35.9	9.20	0.4551
	Midday (1200–1700)	17.2	34.9	4.18	0.0402
	Evening (1700–2100)	23.8	15.6	3.58	0.1768
	Overall	22.1	28.8	3.45	0.2410
Drinking Water (%)	Morning (700–1200)	0.27	0.10	0.165	0.5182
	Midday (1200–1700)	0.01	0.19	0.062	0.1098
	Evening (1700–2100)	0.12	0.49	0.261	0.3685
	Overall	0.13	0.26	0.088	0.3572
Eating Mineral (%)	Morning (700–1200)	0.36	0.02	0.234	0.3647
	Midday (1200–1700)	0.08	0.00	0.049	0.3268
	Evening (1700–2100)	0.21	0.05	0.146	0.4703
	Overall	0.22	0.02	0.049	0.0518

^1^ Treatments: WE toxic endophyte-infected tall fescue; NE novel endophyte-infected tall fescue; Twelve fall-born Angus or Angus cross heifers assigned to each pasture treatment. ^2^ Heifers behavior was captured using Moultrie D-500 timelapse trail cameras (EBSCO Industries, Inc., Birmingham, AL, USA).

## Data Availability

The data presented in this study are available on request from the corresponding author.
